# Effect of 8 days of water-only fasting and exercise on liver, kidney and pancreas functions in middle-aged men

**DOI:** 10.1038/s41598-025-32851-9

**Published:** 2025-12-31

**Authors:** Pilis Karol, Rojczyk Ewa, Petr Valach, Pilis Anna, Dolibog Patrycja, Gabryś Katarzyna, Pilis Wiesław

**Affiliations:** 1 Collegium Medicum Jan Dlugosz University in Czestochowa , Częstochowa, Poland; 2 Faculty of Education, Center of Physical Education and Sport , University of West Bohemia, Pilsen, Czechia; 3 Department of Biophysics Faculty of Medical Sciences in Katowice, Medical University of Silesia, Katowice, Poland; 4 Doctoral School in Social Sciences , Jagiellonian University , Krakow, Poland; 5 Faculty of Medicine, Academy of Silesia in Katowice, Katowice, Poland

**Keywords:** Liver, Kidney, Pancreas, Fasting, Exercise, Men, Biochemistry, Diseases, Physiology

## Abstract

Fasting and physical exercise significantly modify the functioning of the body’s internal organs. The aim of the study was to investigate the simultaneous effect of an 8 days of water-only fast and physical exercise on selected functions of the liver, kidneys and pancreas in middle-aged men. The following parameters were determined in the serum and blood of 18 men before and after 8 days of water-only fasting: aspartate aminotransferase (AST), alanine aminotransferase (ALT), uric acid (UA), total protein (TP), albumin (AL), amylase (AY), ferritin (FR), cortisol (C), β-hydroxybutyric acid (β-HB), hematocrit (Ht) and lactate (LA) under conditions of rest and physical exercise. The daily volume of urine excreted, its specific gravity and the concentration of C and AL in it were also determined. Additionally, the De Ritis index, and AL/CR ratio in urine were calculated. The fasting intervention significantly increased the activity of liver enzymes, the concentration of UA, FR, C, β-HB (p < 0.01) as well as the specific gravity of urine (p < 0.05). On the other hand, it decreased AY activity in serum (p < 0.01) and the daily amount of urine excreted (p < 0.05). Physical exercise increased the concentration of TP, AL (p < 0.01) and LA (p < 0.001). Despite many significant changes determining the function of the liver, kidneys and pancreas, no negative health effects caused by either factor were found. The recorded changes only herald potential adverse health effects that may occur if the impact of fasting and/or physical exercise intensifies.

## Introduction

Questions of the scope and dynamics of changes caused in the human body by strong stress factors, such as fasting or intensive physical exercise, still do not have a comprehensive answer. In addition to the reduction of body weight (including the weight of individual organs), many general and organ changes in the body are observed during fasting^1^ and physical exercise^2,3^. Thus, significant changes in the levels of its enzymes are observed in the liver.

It has been suggested that potential causes of these changes may be acute hypoperfusion^4^, low glutathione levels causing oxidative stress^5^ or autophagy of hepatocytes^6^. In the diagnosis of the functional state of the liver and other organs, in addition to determining the levels of aspartate aminotransferase (AST) or alanine aminotransferase (ALT), the De Ritis index is used^7^. The De Ritis index is considered not only as an indicator of the degree of liver damage^8^ but also as a prognostic indicator of an increased risk of developing cardiovascular disease (CVD) in men, but not in women^9^, acute myocardial infarction^10^, type 2 diabetes^11^, cardiac arrest^12^ and acute heart failure^13^.

Serum albumin concentration may also reflect liver function, as it is the main protein produced by this organ. The concentration of albumin in the blood is treated as an indicator for identifying malnutrition before orthopedic surgery^14^, heart transplants^15^ and it is used in patients undergoing dialysis^16^. However, the prognostic value of the serum albumin level as an indicator of malnutrition is questioned, among others, due to the variability of its concentration in various disease states^17,18^. Moreover, the concentration of albumin in serum increases significantly after physical exercise, which is used in anti-doping control in professional sports competitions^19^.

Ferritin (iron storage) is also a protein found, among others, in the liver, and changes in its concentration in serum may reflect the functional status of this organ. Iron deficiency is the main cause of anemia, whereas excess of this element can lead to impaired cognitive development in children, mediate DNA replication stress^20^, and promote the development of other diseases^21^. Changes in iron metabolism may also cause infectious diseases, cancer and ischemia-reperfusion injuries^22^. It was found that the concentration of iron in the blood decreased significantly in the 48th hour of the ultramarathon run, while the concentration of ferritin increased significantly already in the 12th hour remaining at this elevated level until the 48th hour of the run and even 48 h after its completion^2^.

Ketone bodies are produced in the hepatic metabolism of fatty acids in the process of ketogenesis. Among them, the concentration of β-hydroxybutyrate increases the most during starvation, which seems to be beneficial for the body because it is, among others, an alternative energy substrate for the brain, skeletal muscles, kidneys and heart^23^. An increase in the body’s exercise capacity under the influence of β-hydroxybutyrate supplementation has also been demonstrated^24^ as well as an increase in the concentration of ketone bodies in the blood after a single physical exercise^25^.

It has also known that the liver produces uric acid, which is a product of the breakdown of purines in the body. Abnormal concentration of uric acid in the blood (hyperuricemia) is the result of an imbalance between the production of this compound and its elimination, mainly by the kidneys. The concentration of this acid in the blood increases significantly during fasting or extremely intense physical exercise, which may lead to acute kidney damage^26,27^. After the marathon run, increased concentrations of albumin in urine and creatinine and purine degradation products (hypoxanthine, xanthine) in blood were also observed, which may modify kidney function^26^.

However, moderate-intensity exercise normalizes the increased level of uric acid in the blood^28^ without changing such classic indicators of kidney function as the glomerular filtration rate (GRF) or the amount of proteinuria in patients with chronic kidney disease^29^. It also causes a reduction in the protein to creatinine ratio in urine^30^.

Another organ function of which is significantly changed during intense stress (such as fasting or intense physical exercise) is the pancreas, and one of the markers determining the modified functions of this organ is serum level of amylase. This enzyme affects the ability to digest carbohydrates, which changes the accumulation of glycogen in the body during fasting or prolonged physical exercise^31,32^. However, it is unknown to what extent the activity of pancreatic amylase changes during the simultaneous impact of prolonged fasting and intense physical exercise.

Taking into account the above-presented data, the aim of the present study is to determine the effect of 8 days of water-only fasting and intensive laboratory exercise on the function of the liver, kidneys and pancreas by monitoring changes in selected indicators illustrating the functioning of these organs in middle-aged men.

## Materials and methods

### Participants

Eighteen healthy middle-aged male volunteers participated in the study. Since the volunteers were randomly selected, the researchers had no influence on their age, as there were few of them, and the main criterion for participation in the study was good health and previous fasting experience. Since the volunteers were in good physical and mental condition, it can be assumed that the approximately 30-year age difference between the participants should not significantly affect the quality of the obtained results. Before the study began, they were 60.78 ± 10.60 years old and had the following basic somatic data: body weight (BW − 84.91 ± 14.47 kg), body height (BH − 175.79 ± 7.05 cm), body mass index (BMI − 27.42 ± 4.06 kg/m^2^). Detailed somatic characteristics of the volunteers are presented in Table 1. The men studied practiced yoga recreationally at varying levels of advancement, with some practicing it once a week and others up to six times a week. Three days before the experiment, the energy value of the food consumed was assessed using a questionnaire. It turned out that two individuals were vegetarians, while the remaining 16 volunteers followed a mixed diet. The daily caloric value of both diets was similar, reaching 2.700 kcal.

Before the start of the experiment, they underwent a medical interview and examination. The inclusion criteria were as follows: (1) over 40 years of age, (2) current medical examinations, (3) the ability to perform physical exercise of maximum intensity. The exclusion criteria were as follows: (1) smoking, (2) chronic medication use, (3) consumption of alcohol, stimulants and other psychoactive substances, (4) occurrence of chronic diseases, (5) failure to complete the research procedure. All participants declared, that they had already practiced various periods of fasting, but the last one was at least 6 months prior to the current study. The current study was approved by the Committee for Ethics in Scientific Research of Jan Długosz University in Częstochowa (Poland) – document KE-U/9/2024 from the 4th of June 2024. The study protocol respects principles formulated in the World Medical Association Declaration of Helsinki – Ethical Principles for Medical Research Involving Human Subject.

All participants were informed about the purpose and particular planned steps of the study and gave the written, voluntary informed consent to participate in it. Moreover, they remained under medical supervision during the study period, 3 days before it and 3 days after study completion.

**Table 1:** Age and somatic variables for studied men before and after 8 days of water-only fasting (Mean ± SD).

Variable	Before	After	Significance (*p*) Cohen’s d
	Mean ± SD	Mean ± SD	
Age	60.78 ± 10.60	60.78 ± 10.60	NS 0.00
BW [kg]	84.91 ± 14.47	78.94 ± 13.67	< 0.001 −3.92
BH [cm]	175.79 ± 7.05	175.79 ± 7.05	NS 0.00
BF [%]	24.29 ± 6.63	23.44 ± 6.83	< 0.001 −0.47
BF [kg]	21.09 ± 8.03	19.08 ± 7.72	< 0.001 −1.41
FFM [%]	75.73 ± 6.65	76.60 ± 6.84	< 0.01 0.46
FFM [kg]	63.82 ± 9.40	59.92 ± 8.36	< 0.001 −1.74
TBW [%]	55.44 ± 4.87	56.04 ± 5.01	< 0.001 0.45
TBW [kg]	46.72 ± 6.97	43.84 ± 6.17	< 0.001 −1.79
BMI [kg/m^2^]	27.42 ± 4.06	25.48 ± 3.8	< 0.001 −3.41
BSA [m^2^]	2.01 ± 0.19	1.95 ± 0.18	< 0.001 −4.35

BW-body weight; BH-body height; BF-body fat; FFM-fat free mass; TBW-total body water; BMI-body mass index; BSA-body surface area. p - U Mann-Whitney test result; NS - not significant; ±SD - standard deviation; Cohen’s d – effect size: 0.2- small effect, 0.5- medium effect, 0.8- big effect, > 1.3- very big effect.

### Study protocol

**Fig. 1:**
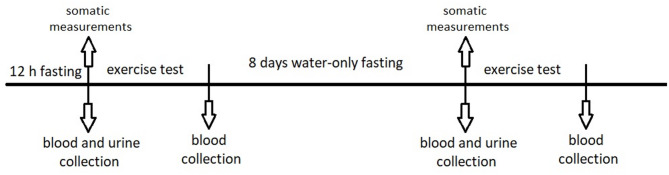
Scheme of the research protocol.

General scheme of research procedures is presented in Fig. 1.

The volunteers were instructed to visit laboratory after a 12-hour long night break from meals and to abstain from alcohol, synthetic medications and dynamic physical exercises two days before the examination. Volunteers reported to the laboratory in the morning and brought with them the entire volume of urine excreted in the 24 h preceding the examination.

Moreover, the age of the examined people was recorded and their body height (BH) was measured. Then, body weight (BW) and its composition were measured using a body composition analyzer (Tanita TBF 300 A - Amsterdam, the Netherlands). The results of this analysis were obtained using bioimpedance. Therefore, prior to the measurement, the volunteers wiped their feet with alcohol to ensure good conductivity with the analyzer electrodes on which they stood. The measurements were taken twice and were completely reproducible. A medically licensed person then collected approximately 10 ml of blood from the antecubital vein. Immediately after blood collection, hematocrit (Ht) was determined. Then, after keeping the blood at room temperature for 10 min, it was centrifuged at 1500 x g for 15 min to obtain serum.

The obtained serum was frozen at −80 °C, and biochemical determinations were performed at a later date. Each determination was performed twice, and the final result was an average to minimize the error. The concentrations of the following parameters were determined in serum: aspartate transaminase (AST), alanine transaminase (ALT), uric acid (UA), total protein (TP), albumin (AL), amylase (AY), ferritin (FR), cortisol (C), and β-hydroxybutyric acid (β-HB). In addition, capillary blood was collected from the finger to determine lactate (LA) concentration.

The daily amount of excreted urine was also examined and its specific gravity as well as the content of AL and creatinine (CR) in it. From the obtained data, the De Ritis index (AST/ALT) in serum and AL/CR ratio in urine were calculated. This was followed by a 0.5 h break during which the volunteers’ heart rate (HR) and oxygen uptake (VO_2_) were determined at rest while sitting. Participants then performed an ergometric exercise test on a bicycle ergometer (Lode Excalibur Sport, Groningen, Netherlands) while wearing a face mask for breathing measurements. The entire cardiopulmonary exercise test was supervised by a physician and conducted by a qualified co-author of this article with 50 years of experience in this field, according to the increasing load protocol used for several decades in our laboratory. The frequency of work, that is, the rate of pedaling was 60 rpm, with an initial load of 30 W, which was systematically increased every 3 min by 30 W, until the maximum individual fatigue (refusal point) was achieved.

The exercise was stopped when the subject reached maximum exercise capacity – that is, when the subject’s VO_2_ and HR stabilized at the maximum level. 3 min after exercise, blood samples were collected once again in order to assess the same biochemical parameters as before test. Blood sampling three minutes after completing physical exercise is commonly used in this type of research, as it is believed that during this period, metabolites and intermediates produced in muscles and other organs have fully recirculated into the bloodstream. During the exercise test and the entire study, the men wore light clothing and athletic shoes that allowed them to comfortably perform the tasks.

The whole abovementioned procedure was repeated after 8 days (192 h) of water-only fasting. During this period, the participants consumed only water *ad libitum* (on average about 3 L/day). During the fast, participants remained in their permanent residences and attended to professional and family obligations. During this time, the men were physically inactive, including practicing yoga, which they had previously practiced, and recorded their body weight daily. Some measurements were taken multiple times during the fast to obtain more accurate results. The reliability of the fasting intervention was assessed by progressive weight loss and increasing serum β-HB concentrations after the fast ended.

Spinreact (Spain) kits were used to determine the levels: AST [the GOT (AST)-LQ; GOT (AST); NADH. Kinetic UV. IFCC rec. Liquid], ALT [the GPT (ALT)-LQ; GPT (ALT); NADH. Kinetic UV. IFCC rec. Liquid], AL (Albumin, Bromcresol green. Colorimetric), AY (Amylase- LQ; α-Amylase; CNPG3. Kinetic. Liquid). FR and C levels were assayed by kit Maglumi Ferritin (CLIA), and Maglumi Cortisol (CLIA), Shenzhen New Industries, Biomedical Engineering Co., Ltd., Shenzhen, China. The concentration of UA in serum and was determined by enzymatic methods using the Abbott’s Architect c System 4000 analyzer. The serum concentration of TP was measured by means of the biuret method using the Architect/Aeroset 4000 device by Abbott. The serum concentration of β-HB was determined enzymatically using the Ranbut diagnostic kit by Randox (UK). Blood lactate concentration was determined using the epoc^®^ blood analysis system (Epocal Inc., Ottawa, Ontario, Canada). Urine specific gravity was determined using the Biomaxima BM URI apparatus. The concentration of AL in urine was measured based on the reaction of the antibody with albumin in the presence of polyethylene glycol and the absorbance of this solution was read at a wavelength of 531 nm. The concentration of CR in urine was based on the Benedict-Behre reaction in which creatinine and 3,5 dinitrobenzoic acid form a colored solution complex at high pH, the absorbance of which was measured at a wavelength of 531 nm. Quantitative concentrations of both substances were determined on the basis of calibration curves of absorbance as a function of their concentrations. HR was measured using a transmitter (Polar) placed on the chest of the subject, which was part of a quick breath analyzer (Ergo Card - Belgium) used to determine VO_2_.

### Statistical analysis

All statistical analysis were performed with STATISTICA version 13.3 (TIBCO Software, Palo Alto, CA, USA). The Shapiro-Wilk test was used to check the normality of the distribution. Because the data were non-normally distributed, differences between the results (before and after 8 days of water-only fasting, before and after vigorous exercise) were calculated using Friedman’s analysis of variance with the Dunn-Bonferroni post hoc test. Statistical comparisons of somatic date, exercise tests and variables determined in urine were performed using the Mann-Whitney U formula. To calculate the effect size, Cohen’s d and η² were used. P values < 0.05 are considered statistically significant. The sample size necessary to demonstrate statistical significance of variables recorded before and after fasting was estimated at *p* = 0.05 and the power of the test was 0.8, which amounted to 12 patients. The values obtained ​​were subjected to statistical analysis using SPSS Statistics 20. Sample size analysis was performed using Statistica v 13.3 (TIBCO) based on the results of the pilot study.

## Results

Statistical analysis showed that at the final load, HR max was not statistically different both before and after the fasting intervention (*p* > 0.05; Cohen’s d = 0.14), as in the first study the heart rate was 152.9 ± 17.1 bpm and in the second study 150.3 ± 21.1 bpm.

**Fig. 2:**
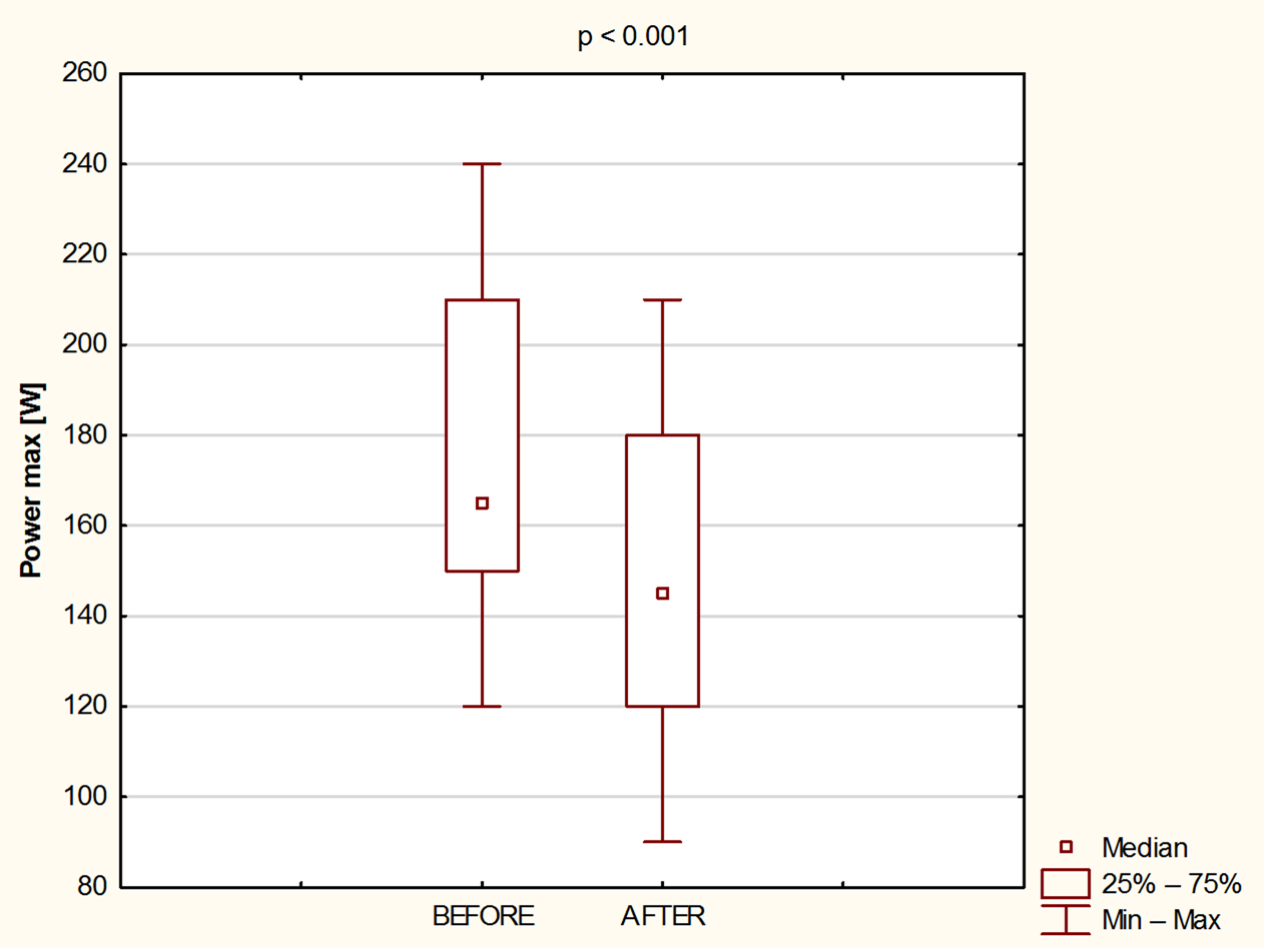
Absolute values ​​of maximum power before and after 8 days of water - only fasting.

At the final load, absolute Pmax values ​​(Fig. 2) were reduced (*p* < 0.001; Cohen’s d = 0.71) by the fasting intervention from 174.7 ± 40.6 W to 147.2 ± 41.6 W).

**Fig. 3:**
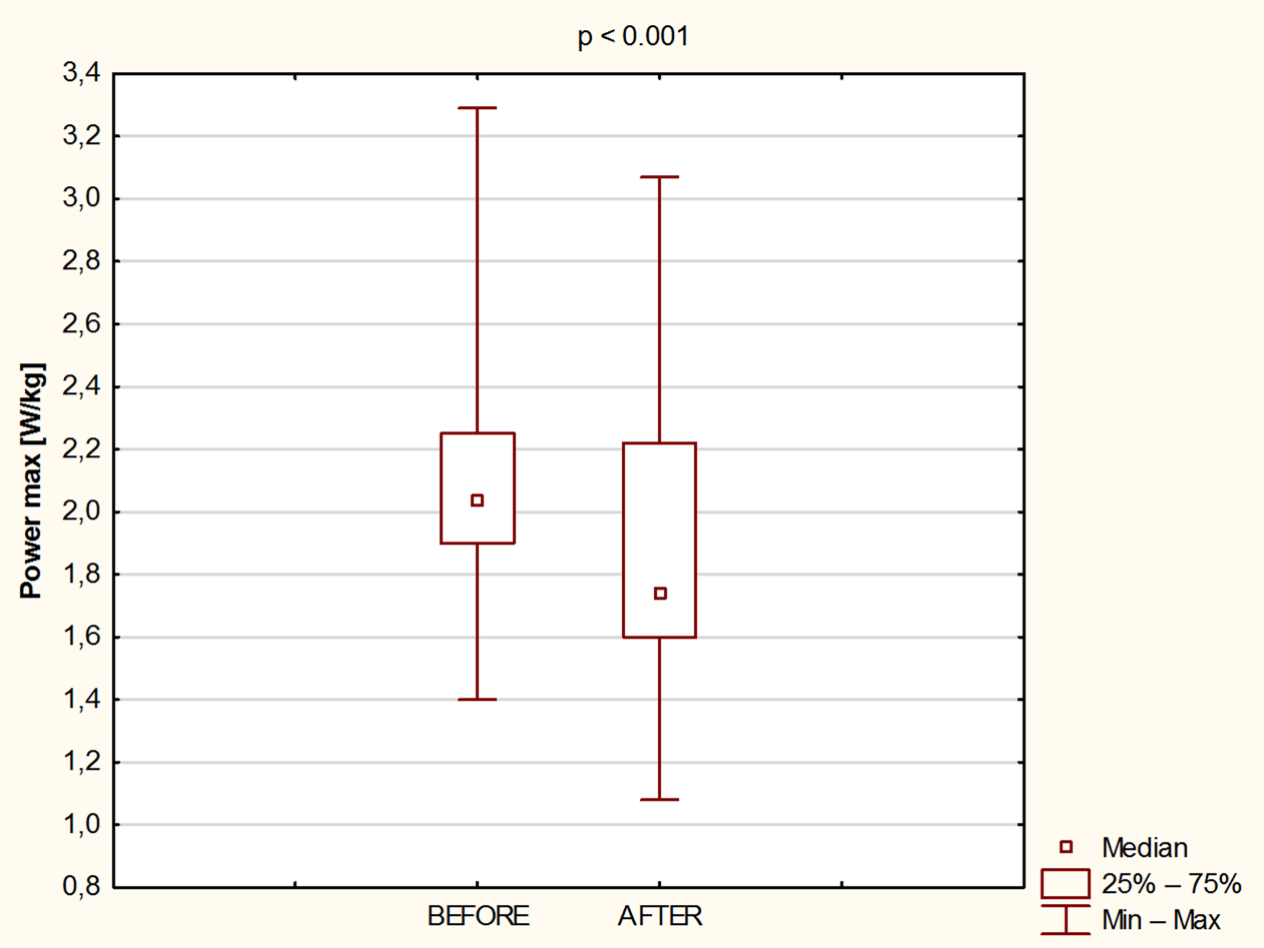
Relative values ​​of maximum power before and after 8 days of water - only fasting.

Relative Pmax was also significantly reduced (*p* < 0.001; Cohen’s d = 0.44) after the fasting intervention from 2.1 ± 0.4 W/kg to 1.9 ± 0.5 W/kg (Fig. 3).

**Fig. 4:**
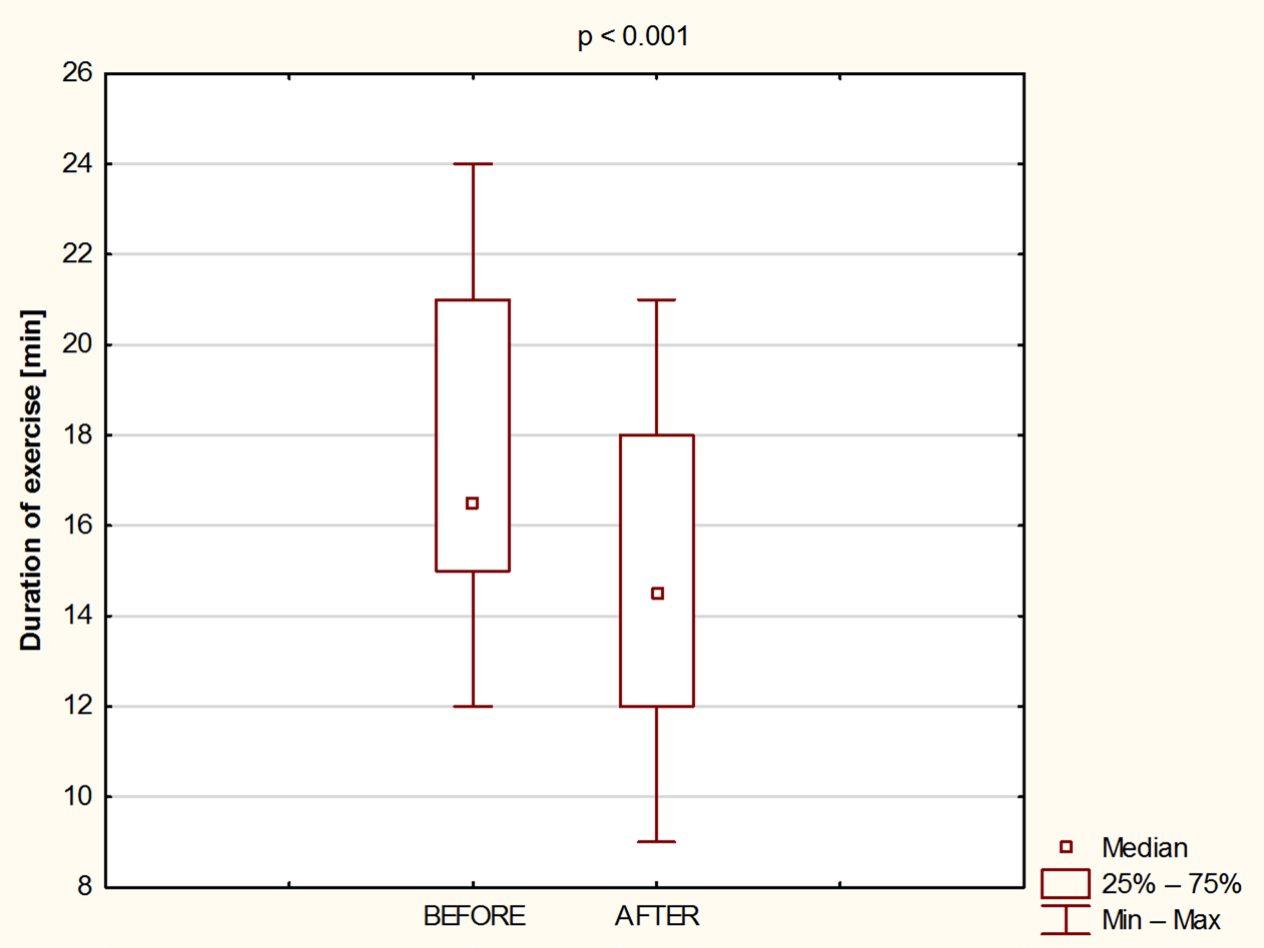
Duration of exercise before and after 8 days of water - only fasting.

Also, the duration of the exercise test was significantly shortened (*p* < 0.001; Cohen’s d = 0.72) after 8 days of water-only fasting from 17.4 ± 4.1 min to 14.7 ± 4.2 min (Fig. 4).

**Fig. 5:**
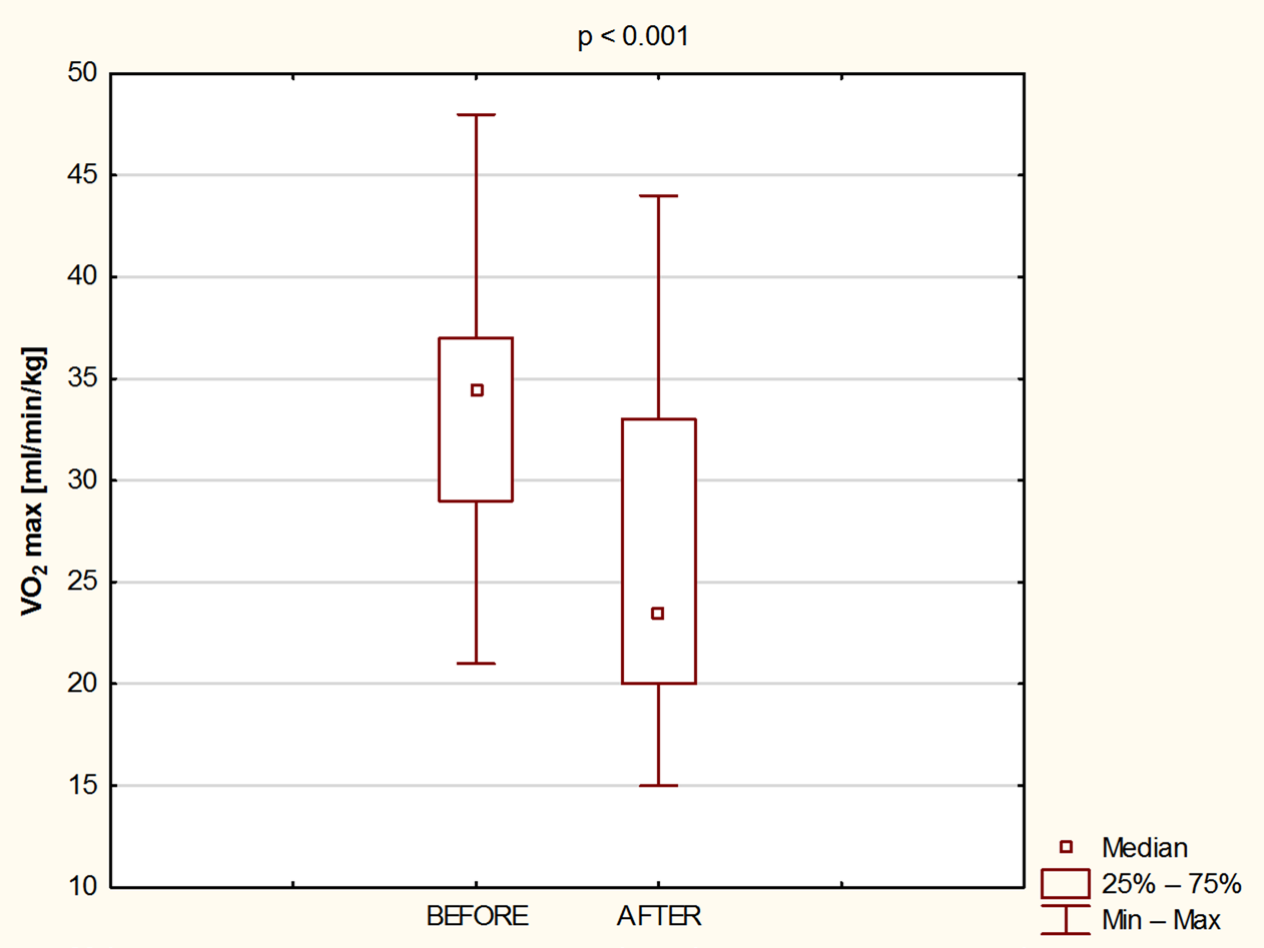
Maximal oxygen uptake before and after 8 days of water - only fasting.

There was also a significant reduction in VO_2_max (*p* < 0.001; Cohen’s d = 1.05) after the fasting intervention from 33.4 ± 7.0 ml/min/kg to 25.8 ± 7.5 ml/min/kg (Fig. 5).

Table 2 shows changes in biochemical indicators before and after 8 days of water-only fasting both at rest and after exercise tests.

**Table 2:** Response of serum biochemical parameters to fasting and exercise (Mean ± SD).

Parameters	Rest before fasting (1)	Rest after fasting (2)	Exercise before fasting (3)	Exercise after fasting (4)	η²
AST [U/l]	19.83 ± 4.46	58.72 ± 37.37**	24.00 ± 5.06	60.56 ± 32.84^##^	^0.554^
ALT [U/l]	10.39 ± 5.13	36.06 ± 31.26**	13.28 ± 5.47	38.89 ± 29.87^#^^#^	^0.269^
AST/ALT	2.19 ± 0.81	1.95 ± 0.73	2.01 ± 0.78	1.88 ± 0.61	0.026
UA [mg/dl]	6.23 ± 1.24	14.42 ± 1.79**	6.43 ± 1.35	14.90 ± 1.89^##^	^0.0879^
β-HB [mmol/l]	0.29 ± 0.18	4.65 ± 0.61**	0.21 ± 0.14	4.06 ± 0.64^##^	^0.0956^
AY [U/l]	66.67 ± 24.50	49.83 ± 19.34**	69.22 ± 24.61	52.11 ± 19.35^##^	^0.137^
FR [ng/ml]	62.60 ± 37.83	140.11 ± 79.27**	69.78 ± 42.17	155.12 ± 86.27^##^	^0.289^
C [ng/ml]	309.19 ± 92.70	479.33 ± 123.54*	320.71 ± 111.68	511.61 ± 137.27^##^	^0.389^

AST - aspartate transaminase; ALT - alanine transaminase; UA - uric acid; β-HB - beta-hydroxybutyric acid; AY - amylase; FR - ferritin; C - cortisol; ±SD - standard deviation; Dunn-Bonferroni comparisons: different than (1) ** - p < 0.01, * - p < 0.05; different than (3) ## - p < 0.01; η² - effect size: ≈ 0.01 – small effect, ≈ 0.06 – medium effect, ≈ 0.14 – big effect

Statistically significant differences were found for concentrations of: AST, ALT, UA, β-HB, AY, FR and C. The fasting intervention resulted in a significant increase in the levels of AST, ALT, UA, β-HB, FR, C. Concentration of creatinine and AST/ALT ratio did not change and the AY decreased significantly (*p* < 0.01) after 8-day fasting.

The exercise tests used had no effect on the parameters listed in Table 2 (*p* > 0.05). However, after the exercise test, statistically significant higher values of TP, AL, Ht and LA in serum were found, but fasting alone did not cause any significant changes. (Table 3).

**Table 3:** Response of serum proteins and hematocrit to fasting and exercise (Mean ± SD).

Parameters	Rest before fasting (1)	Rest after fasting (2)	Exercise before fasting (3)	Exercise after fasting (4)	η²
TP [g/l]	72.30 ± 5.40	74.12 ± 4.27	79.08 ± 5.94**	79.82 ± 5.56^##^	0.276
AL [g/l]	51.11 ± 3.81	52.28 ± 3.23	61.00 ± 16.96**	61.0 6± 13.96^##^	^0.163^
Ht [%]	43.61 ± 3.40	42.78 ± 3.98	47.56 ± 3.11***	46.94 ± 3.89^###^	^0.286^
LA [mmol/L]	1.23 ± 0.55	1.59 ± 0.48	8.88 ± 2.12***	7.07 ± 1.61^###^	^0.862^

TP - total protein; Al - albumin; Ht - hematocrit; LA - lactate; ±SD – standard deviation; Dunn-Bonferroni comparisons: different than (1) ** - p < 0,01, *** - p < 0,001; different than (2) ## - p < 0.01, ### - p < 0.001; η² - effect size: : ≈ 0.01 – small effect, ≈ 0.06 – medium effect, ≈ 0.14 – big effect

Table 4 shows changes in renal function and urine parameters after 8 days of water-only fasting. Under these conditions, only a reduction in the amount of urine excreted and an increase in its specific gravity were observed (*p* < 0.05). The remaining variables included in this table have not changed.

**Table 4:** Changes in kidney function and urine after 8 days of water-only fasting (Mean ± SD).

Parameters	Rest before fasting	Rest after fasting	Significance (*p*)	Cohen’s d
Volume [ml/24 h]	1741.67 ± 1059.59	1327.78 ± 609.06	< 0.05	−1.4
Volume [ml/min]	1.21 ± 0.74	0.92 ± 0.42	< 0.05	−0.77
Specyfic gravity [g/ml]	1.019 ± 0.004	1.023 ± 0.003	< 0.05	0.69
Creatinine [g/24 h]	1.81 ± 0.58	1.61 ± 0.50	NS	−0.25
Creatinine [mmol/l]	14.71 ± 7.30	16.70 ± 8.24	NS	0.26
Albumine [mg/24 h]	12.69 ± 8.96	15.01 ± 10.98	NS	0.42
Albumine/Creatine [mg/g]	7.52 ± 4.99	9.40 ± 6.64	NS	0.18

±SD – standard deviation; p - U Mann-Whitney test result; NS – not significant; Cohen’s d – effect size: 0.2- small effect, 0.5- medium effect, 0.8- big effect, > 1.3- very big effect.

## Discussion

Significantly increased cortisol concentrations in the serum of the studied men after 8 days of water-only fasting and maximum physical exercise indicate the stimulation of a strong stress reaction, which was activated mainly by the fasting intervention. This effect concerned the functioning of the liver, kidneys and pancreas. This form of chronic 8-day fasting has been used repeatedly in our previous studies and did not cause adverse psychological changes^33^. Simultaneously, under these conditions, we observed an improvement in the urogenital system^34^ in middle-aged men. The current study also indicates that the 8-day fasting intervention resulted in a reduction in maximal power developed during an ergometric exercise test, a shortening of its duration, and a reduction in VO_2_max. The same changes occurred during the 7-day fasting described by Kolnes et al.^35^. The authors attribute these changes to a reduction in carbohydrate oxidation while maintaining the level of oxidative enzymes in muscles during a high-intensity ergometric test under fasted conditions. Our results do not support conclusions about the effect of fasting on carbohydrate utilization in the glycolytic process. This finding stems from the lack of significant differences in blood lactate concentrations between fasting and physiologically fed conditions. Only trends toward increased lactate concentrations at rest and lower concentrations during maximal exercise were observed under these conditions. The significant reduction in oxygen uptake and the tendency to achieve lower blood lactate concentrations at the final ergometric load (VO2peak) during fasting observed in our study are also likely related to lower maximum power output and shorter exercise test duration.

Despite achieving maximum/peak values ​​in heart rate, power output, and oxygen uptake before and after 8 days of water-only fasting, it should be concluded that the exercise test used in our study did not induce an enhanced stress response. This is confirmed by the lack of post-exercise increases in serum cortisol concentrations both before and during fasting.

The maximum intensity exercise test used in our research did not contribute to the formation of an increased stress reaction. These data differ from the indications of other authors who observed an increase in serum cortisol concentration during maximum intensity exercise, performed on an ergometer and a treadmill, regardless of nutritional status^36^. In other studies, a decrease in serum cortisol concentration was observed after 60 min of mild exercise, suggesting that such a stimulus may be a factor mitigating the stress response^37^.

### The influence of fasting and physical exercise on liver function

To assess liver function, it is important to determine the levels of aminotransferases such as AST and ALT, the activity of which in our studies significantly increased under the influence of 8 days of water-only fasting, without the participation of physical exercise. This is an important observation because their levels are not only biomarkers of damage to this organ, but also indicate the risk of other diseases^38^.

Interestingly, fasting and then refeeding procedure caused liver damage in mice^39^. Histological changes indicating microvesicular steatosis of the liver, were also observed in rats after 24 h of fasting, despite the lack of significant differences in liver enzyme concentrations^40^. Moreover, a similar experiment, but conducted ex vivo on rat liver, showed that after 18 h of fasting, the concentration of AST and ALT, as well as other cytolysis markers, increased^41^. Significantly increased activities of both AST and ALT in our experiment suggest an adverse effect of 8 days of water-only fasting on the functional state of the liver, but the lack of changes in the De Ritis index did not confirm this. The values of this indicator in our research were around 2, which was not drastically high compared to the maximum levels. However, it is also confirmed, that a low-calorie diet combined with a moderate exercise program effectively lowers liver enzyme levels^42^. Similarly, in patients suffering from severe β-thalassemia who practiced intermittent Ramadan fasting, liver enzyme levels showed a decreasing trend^43^. It is important to note, however, that the adaptive capacity of humans and some animals (penguins, bears, seals) to starvation differs, as these animals can survive for months without food or water. This difference compared to humans stems from distinct biochemical adaptations in lipid, carbohydrate, and especially protein metabolism that allow these animals to survive^44^.

There are significant controversies regarding the determination of liver function by monitoring changes in serum protein level (including albumin concentration) during fasting or physical exercise. In our studies, serum albumin concentration did not change after the fasting intervention. Similarly, other studies have also shown that in healthy people, serum albumin levels remained within the accepted limits until extreme starvation lasting more than 6 weeks or reaching a BMI < 12^45,46^. However, controversial results were obtained after 3 days of fasting, when the concentration of albumin and total protein in the serum of 18–22-year-old students increased significantly^47^.

Despite these controversial data, it is worth recording the level of albumin in serum, because this protein has an antioxidant effect during fasting^48^. Recent studies using proteomic profiles, which analyzed changes in the concentrations of approximately 3,000 plasma proteins before, during, and after a 7-day fast, showed that systemic changes in the body begin only after 3 days of such an intervention. After this time, potential positive health benefits were observed in conditions such as rheumatoid arthritis and heart disease, as well as adverse effects^49^. However, intermittent fasting lasting less than 48 h did not demonstrate such beneficial effects compared to longer-term caloric restriction^50^.

It should be noted that the serum albumin concentration after the exercise test in our study increased significantly both before and after the fasting intervention. It should be assumed, that this increase results from a post-exercise, short-term reduction in plasma volume, confirmed by a significant increase in hematocrit determined after the end of exercise tests, which was also confirmed in other studies^19^. There is also data indicating that physical exercise may modify the concentration of albumin in skeletal muscles, the heart or the liver in conditions of changes in the prooxidant/antioxidant balance or in the muscle mass^51,52^.

The serum ferritin content may also reflect the functional state of the liver, because this protein is stored in this organ. Its concentration increased significantly under the influence of 8 days of water-only fasting, but the exercise test did not modify these changes. Although the observed changes in ferritin concentration reached significant, almost 2,5-fold increases, these values were within the limits of accepted physiological norms and cannot be an indicator of either a positive or negative impact of the fasting intervention on the functional state of the liver^21,22^.

In the present study, we also observed a significant, over 2-fold increase in serum uric acid concentration after 8 days of water-only fasting, which was not modified by the exercise test used. Although the increase in uric acid concentration did not cause any visible health complications in the examined volunteers, it should be considered as an unfavourable effect. Uric acid acts as a strong antioxidant, which determines approximately 60% of the antioxidant potential of human plasma^53^. However, when it enters the cellular environment, it activates oxidative stress, which affects multi-organ negative changes^54^.

The liver is also an organ where ketone bodies are produced, and the concentration of one of them (β-HB), increased several times as a consequence of the fasting intervention. It is known, that during prolonged fasting also other ketone bodies are produced and in extreme cases their global concentration in the blood may reach ~ 25mM. This increase is associated with depletion of bicarbonate stores to almost zero^55^, which may contribute to the transition of ketonemia to an unfavorable condition for the body called the state of ketoacidosis. Ketonemia may also be generated by chronic physical exercise^56^, which (combined with prolonged fasting) may be dangerous to health, especially in people with any liver disease^57^. However, the exercise test used in our study, although characterized by maximum intensity, was not long enough to additionally increase the concentration of ketone bodies. Therefore, due to the possibility of ketonemia turning into ketoacidosis, it is suggested not to extend the fasting intervention significantly beyond 8 days, despite its undoubted advantages, and not to undertake long-term, strenuous physical efforts during this time.

### Kidneys function under conditions of fasting and physical exercise

In our study, we monitored the functional efficiency of the kidneys during the fasting intervention by measuring the urinary albumin to creatinine ratio. This index did not confirm the negative impact of an 8 days of water-only fasting on kidney functions, because this ratio did not change and did not exceed physiological norms^58^. Stabilized values ​​of this index suggest a good functional state of the kidneys of the volunteers included in the presented studies. However, it is worth mentioning that in the conditions of increased AST and ALT levels observed in our study after 8 days of water-only fasting, visceral and systemic vessels may dilate and renal vessels may narrow, which can potentially result in impaired function of this organ^59,60^.

Moreover, it has been shown that hyperuricemia (also observed in our study) can cause significant changes in systemic and renal hemodynamics, which may result in the loss of autoregulatory kidneys function^54^. This potentially generated hypertension, insulin resistance, obesity, hypertriglyceridemia and metabolic syndrome^61^.

It should also be noted that 8 days of water-only fasting also resulted in a decrease in the volume of excreted urine and an increase in its specific gravity. However, the scope of both changes, although statistically significant, was within the limits of accepted norms for healthy people.

Although in our study we observed an increase in AST and ALT levels, a decrease in urine output and an increase in urine specific gravity, the classic indicator of kidney function, such as the urinary albumin to creatinine ratio, did not change under the influence of fasting. Globally, such changes do not indicate the development of pathological reactions in the kidneys initiated by the fasting intervention. However, to maintain safe kidney functioning (to avoid possible health complications), it is suggested that an 8 days of water-only fasting period should be considered as extremely long.

### Pancreatic function under conditions of fasting and physical exercise

The role of the pancreas in the present experiment can be concluded based on changes in the amylase activity, which was significantly reduced as a result of 8 days of water-only fasting. This change may be associated with the depletion of carbohydrate reserves that occur during fasting^62^. Kolnes et al.^35^ also demonstrated that after 7 days of fasting, in addition to an approximately 50% reduction in muscle glycogen, there is a reduction in the ability of skeletal muscle to oxidize carbohydrate. This effect may be necessary to prevent hypoglycemia during fasting, but it may also prevent increased aerobic energy turnover during exercise. These authors suggested that these metabolic trade-offs may be responsible for the reduction in maximal aerobic capacity during fasting, a finding also confirmed in our study. Literature data indicate that during intense endurance exercise lasting several hours, amylase activity decreased along with a decrease in carbohydrate reserves^63^. Therefore, the lack of changes in the activity of this enzyme even after very intense aerobic exercise lasting only a dozen or so minutes in our study is a normal reaction, which indirectly indicates the lack or slight reduction of carbohydrate resources in this period^63^.

It is worth mentioning that endurance training performed over a period of 4 weeks or longer caused an adaptive increase in amylase activity in rats, which was associated with an increased ability to digest carbohydrates and, as a result, with a greater accumulation of glycogen in the muscles, which leads to an increased exercise capacity^31,32,64^.

To sum up, it should be stated that the determination of the level of amylase in serum has substantive justification, as it indirectly reflects the size of the body’s carbohydrate reserves, which are significantly modified by both fasting and intensive physical exercise. In our study, the first of these factors (fasting) played an important role in reducing the activity of amylase.

### Strength and limitation

Important factors limiting the analysis of results and its discussion in this study are the relatively small group of participants and significant differences between the protocol used in our study and those found in the literature. Daily fluctuations of the concentrations of some parameters recorded in our study additionally complicates the interpretation of the results. To eliminate it, more measurements should be performed (at different times of the day). It would also be necessary to examine the long-term effects (especially of the fasting intervention used) e.g. after a period of 2 weeks. We also consider the lack of biochemical testing during the fasting period to be a significant limitation of this study. Such testing, in addition to its cognitive effects, would have allowed for more secure monitoring of the fasting period administered to the volunteers participating in this experiment. To ensure safety and maintain the relative well-being of these men, only blood pressure, heart rate, body temperature, and body weight were recorded during fasting. Finally, the experimental protocol could also be improved by checking whether lifestyle influences the results, for example by dividing subjects into a physically active and inactive group. Future research should also consider different patterns of fasting and exercise.

Such expansion of research protocol would provide a broader picture of the impact of caloric restriction and/or fasting combined with physical exercise on the human body health parameters.

## Conclusions

Detailed analysis of the results showed that the increased levels of AST, ALT and ferritin, although statistically significant, were within or slightly exceeded physiological norms. Thus, they are only a warning signal against long-term maintenance of a state of fasting, which may be unfavorable for the liver or kidneys. Significant elevation in β-HB and uric acid concentrations clearly indicate adverse metabolic changes, both for the liver, kidneys, and the entire body, resulting from the fasting intervention. On the other hand, normal values of the albumin/creatinine ratio in urine and constant values of the AST/ALT ratio in serum indicate a stabilized functional state of the kidneys and liver in the described conditions. Moreover, a strong effect of the fasting intervention was observed in relation to amylase concentration decrease, indicating a significant reduction in carbohydrate stores in the men tested under these conditions. No effect of fasting on the intensity of the glycolysis process was observed.

The dominant, although transient, effect of physical exercise was observed only in case of albumin and total protein concentrations in the blood – these parameters increased after exercise.

In summary, the 8 days of water-only fasting and concurrent physical exercise caused significant stress on the body, but no lasting negative health changes were observed. The obtained results may be useful for people conducting prolonged health fasts, but medical supervision is recommended during their duration.

## Data Availability

The datasets used and/or analysed during the current study are available from the corresponding author on reasonable request.

## Abbreviations

AST: Aspartate transaminase
ALT: Alanine transaminase
UA: Uric acid
TP: Total protein
AL: Albumin
AY: Amylase
FR: Ferritin
C: Cortisol
β-HB: Beta-hydroxybutyric acid
Ht: Hematocrit
CVD: Cardiovascular disease
GRF: Glomerular filtration rate
BW: Body weight
BH: Body height
BF: Body fat
FFM: Fat free mass
TBW: Total body water
BMI: Body mass index
BSA: Body surface area
